# Management of pleural relapse after breast cancer resection in a middle-aged man: a case report

**DOI:** 10.3389/fonc.2026.1759603

**Published:** 2026-03-09

**Authors:** Wei Wang, Nanlin He, Jintang Tu, Weijia Huang, Xiaoming Qiu

**Affiliations:** 1 Lung Cancer Center/Lung Cancer Institute, West China Hospital, Sichuan University/West China School of Nursing, Chengdu, China; 2 Operating Room of Anesthesia Surgery Center, West China Hospital, Sichuan University, Chengdu, China; 3 Lung Cancer Center/Lung Cancer Institute, West China Hospital, Sichuan University, Chengdu, China; 4 Department of Thoracic Surgery, West China Hospital, Sichuan University, Chengdu, China

**Keywords:** breast cancer recurrence, case report, male breast cancer, palliative surgery, pleural metastasis

## Abstract

Male breast cancer (MBC) is a rare breast carcinoma subtype with limited available data to fully delineate its recurrence patterns and guide evidence-based therapeutic strategies. We report a rare case of pleural relapse in a 59-year-old male patient following a radical resection of right breast cancer. Initially diagnosed with pathological stage IIIB disease, the patient then underwent adjuvant chemotherapy, radiotherapy, and endocrine therapy. During postoperative surveillance, persistent nodular pleural thickening along the interlobar fissures of the right lung was detected. Thereafter, the patient underwent pulmonary nodule resection and systematic lymph node dissection, with electrocautery-assisted resection or excision of all visible pleural nodules, followed by an immediate platinum-based intrapleural perfusion chemotherapy. Histopathological and immunophenotypic analyses confirmed metastatic breast carcinoma. Adjuvant therapy with abemaciclib and letrozole was initiated, and no recurrence was observed during the 27-month postoperative follow-up. Taken together, our findings underscore the importance of screening for solitary pleural metastasis at initial diagnosis and during follow-up for MBC and support a potential role for palliative surgical resection in locoregional MBC recurrence to achieve durable disease control and prolonged survival, providing a feasible treatment option for carefully selected patients.

## Introduction

Male breast cancer (MBC), a rare malignancy accounting for approximately 1% of breast carcinomas, typically manifests as a painless, palpable breast mass with potentially poor mobility (1, 2). Compared with female breast cancer (FBC), MBC usually presents with a higher median age at diagnosis (67 vs. 62 years) and a higher proportion of advanced-stage diagnoses (40% vs. 14%), which is associated with compromised overall survival regardless of tumor staging (1, 3). MBC is associated with a higher incidence of lymphatic or distant metastasis across the various molecular subtypes (human epidermal growth factor receptor 2 [HER2], estrogen receptor [ER], and progesterone receptor [PR]), and it ranges a higher proportion of the HER2-negative subtype with hormone receptor (HR; including ER and PR)-positive (male vs. female, 90% vs. 71%) (1, 4). Frequent sites of distant metastasis include the bone (60%–75%), lung (32%–37%), liver (32%–35%), and brain (10%) for recurrent breast cancer, whereas solitary pleural metastasis occurs less commonly (5). Treatment strategies for male patients are mainly based on FBC studies due to the low incidence of MBC and limited evidence, which may overlook the underlying discrepant pathogenesis. Here we report a rare case of a 59-year-old male patient diagnosed with pleural relapse after a radical resection of breast cancer, and intend to demonstrate that surgical intervention could be an alternative treatment for locoregional recurrent diseases.

## Case presentation

**Figure 1:**
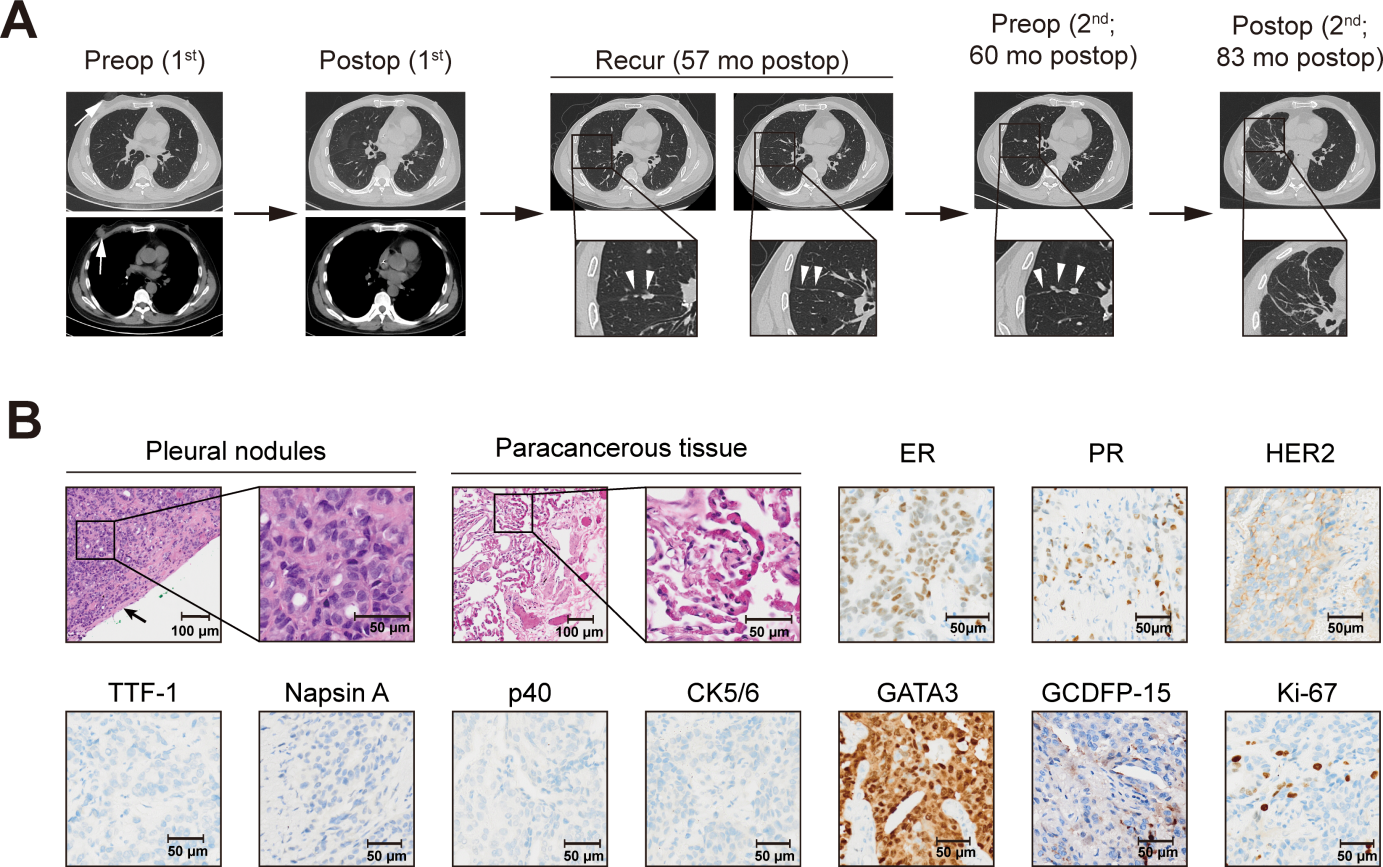
Diagnosis and treatment timelines based on imaging findings **(A)** and pathological characteristics of pleural metastasis **(B)**. **(A)** Chest computed tomography showing the right breast cancer before mastectomy (the white arrows indicate breast cancer), recurrence on the right lung pleura (the white triangles indicate the pleural metastases), progressive enlargement, and follow-up 27 months postoperatively. **(B)** Hematoxylin–eosin staining revealed that the tumor was adjacent to the pleura (the black arrow indicates the pleura) but it did not infiltrate the paracancerous tissue; immunohistochemical staining of the pleural metastasis (surgical specimen) was positive for ER, PR, GATA3, and GCDFP-15, with partial positivity for Ki-67 (30%) and HER2, while negative for TTF-1, Napsin A, p40, and CK5/6. ER, estrogen receptor; GATA3, GATA binding protein 3; GCDFP-15, gross cystic disease fluid protein 15; HER2, human epidermal growth factor receptor 2; PR, progesterone receptor; TTF-1, thyroid transcription factor 1.

A 59-year-old man first noticed a soft and palpable mass in his right breast with a size of 1 cm × 1 cm in 2015, but sought no medical attention. On subsequent chest computed tomography (CT) imaging 3 years later, the lesion had progressively enlarged to 2 cm × 2 cm × 1.5 cm (**Figure 1A**), exhibiting increased radiodensity and accompanied by mild dull pain with involvement such as skin ulceration in the ipsilateral chest wall. The patient’s preoperative Eastern Cooperative Oncology Group performance status was 1. Then, he received a right simple mastectomy with axillary lymph node dissection at this medical center, with the histopathological examination confirming invasive ductal carcinoma. Immunohistochemical (IHC) analysis revealed positive expression of ER, PR, E-cadherin, and D2-40, while CK5/6 and p63 were negative. The proliferation marker Ki-67 showed a labeling index of 30%. Fluorescence *in situ* hybridization (FISH) confirmed HER2 negativity with no evidence of gene amplification. Of the 18 axillary regional lymph nodes examined during the surgery, two were confirmed to have metastasized upon histopathological assessment. The final stage was pathological stage IIIB, pT4bN1aM0 (American Joint Committee on Cancer [AJCC] Cancer Staging Manual, 8th Edition). The patient completed four cycles of EC chemotherapy (epirubicin and cyclophosphamide), followed by four cycles of T chemotherapy (docetaxel). Subsequent therapy included radiotherapy and 3 months of tamoxifen.

At 3 months before admission, a chest CT showed multiple nodules in the horizontal fissure and the oblique fissure of the right lung, with the largest one (1.0 cm × 0.8 cm) localized in the oblique fissure of the right lung (**Figure 1A**). A follow-up CT at 9 days pre-admission demonstrated persistent nodular pleural thickening along the interlobar fissures, with the largest nodule (1.0 × 0.8 cm) showing stable dimensions compared to prior imaging. An axillary evaluation revealed architectural distortion in the right axilla without significant bilateral lymphadenopathy. In consideration of long-term chemotherapy and hormone therapy, the patient requested surgical intervention for the pleural metastasis. The patient reported occasional alcohol consumption with unclear frequency and denied tobacco use or substance abuse. No family history of breast cancer was documented. The preoperative assessment confirmed an adequate performance status and the absence of significant comorbidities.

After a multidisciplinary assessment and comprehensive informed consent detailing the risks and benefits, the patient underwent resection of the lateral basal segment of the right lower lobe and the lateral segment of the middle lobe, along with systematic lymph node dissection. All macroscopically visible pleural nodules underwent *en bloc* resection or electrocautery-assisted excision, followed by immediate intrapleural perfusion with a platinum-based chemotherapeutic agent. No pleural effusion was observed intraoperatively. Gross examination of the resected lung tissue revealed multiple grayish-white, firm pleura-based nodules adjacent to the pleura. IHC staining of formalin-fixed paraffin-embedded tissue samples confirmed metastatic carcinoma with strong positivity for ER, PR, and GATA binding protein 3 (GATA3), partial positivity for HER2, and gross cystic disease fluid protein 15 (GCDFP-15) (**Figure 1B**). FISH showed no HER2 amplification. Lymph nodes from stations 7, 9, and 10 were pathologically negative for metastasis. The histopathological and immunophenotypic features of the pleural nodules were consistent with primary breast carcinoma, confirming the diagnosis of pleural metastasis from breast cancer. Adjuvant treatment was administered with concurrent abemaciclib and letrozole until the follow-up 27 months after surgery, and the patient reported a marked improvement in quality of life during follow-up, with no evidence of disease recurrence detected (**Figure 1A**).

## Discussion

MBC is a rare malignancy with limited studies characterizing its recurrence patterns, among which pleural recurrence remains particularly underreported, as are effective therapies (1, 6, 7). In this case, we presented an unusual instance of solitary pleural recurrence without pleural effusion after surgical resection of breast carcinoma. In our opinion, oligoprogression and solitary pleural disease are limited metastatic diseases, representing distinct biological subsets of limited invasiveness compared to widespread metastatic dissemination (8). Hence, we administered local consolidative therapy to the patient after resecting the visible pleural deposits and conducting electrocautery followed by intrapleural perfusion, to achieve superior disease control. We described the case of a male patient who developed solitary pleural recurrence after curative surgical resection of breast cancer and delineated potentially effective therapeutic approaches for pleural metastatic deposits in this rare clinical setting.

The impact of gender differences on breast cancer survival has been overestimated, and the prognosis is comparable between male and female patients after adjusting for age at diagnosis, comorbidities, and tumor staging (1, 9). However, MBC is associated with older age at diagnosis and a higher incidence of metastasis, with distant metastasis serving as an independent prognostic factor; meanwhile, the prognosis for metastatic diseases is comparable between MBC and FBC (2, 10). The thorax is a common site for initial breast cancer metastasis; however, solitary pleural metastasis remains rarely reported (11–13). A potential biological tendency exists for right breast cancer to metastasize to the ipsilateral pleura of the right thorax, similar to the tendency of lung cancer to metastasize to the ipsilateral breast, as previously reported (14). Additionally, MBC exhibits a higher proportion of hormone receptor (HR)-positive, HER2-negative tumors compared to FBC. Consequently, for HR-positive cases with aggressive progression, we administered adjuvant chemotherapy combined with tamoxifen as part of the treatment regimen.

Systemic therapy, including endocrine therapy and chemotherapy, remains the standard of care for metastatic or recurrent breast cancer, while the role of metastasectomy remains controversial, particularly among oncologists (15, 16). Although previous studies have explored the potential survival benefits of surgical resection for thoracic metastases, definitive evidence supporting its advantages remains inconclusive (15, 17). Locoregional treatment, including metastasectomy, may serve as a viable option for patients with oligometastatic disease, with some evidence suggesting improved survival outcomes (15, 18). In this case, stereotactic ablative radiotherapy (SABR) was not initially considered due to the presence of multiple pleural deposits despite its established association with improved overall survival in selected settings (19). While the clinical benefit of pleural deposit resection has not been well described, the quality of resection might not be a significant prognostic factor as it is in the early-stage diseases. Hence, we proposed that aggressive treatment of pleural metastases could lead to local disease control and improved long-term survival although the therapeutic role has not been well proven (20). Furthermore, removal of the visible metastases in advance could prevent regional dissemination and delay disease progression, hence potentially reducing metastatic burden (15). Beyond its therapeutic implications, surgical intervention may help confirm the pathological diagnosis, particularly given that 34%–75% of suspected lung metastases in breast cancer patients are indeed metastatic lesions (21, 22).

Notably, solitary pleural metastasis, a rarely reported phenomenon in MBC, should be carefully considered during both initial evaluation and treatment decisions. Nevertheless, favorable outcomes for individuals with hormone receptor-positive male breast cancer should be carefully interpreted and generalized, with attitudes toward palliative surgery contingent on the patient’s informed wishes. In summary, the present work briefly outlines the potential therapeutic role of palliative surgical intervention in the management of locoregional MBC recurrence. This approach could deliver durable disease control and extended survival and thus offers a valuable supplementary treatment option for rigorously selected patients, complementing standard systemic therapy. To further refine therapeutic strategies and improve long-term clinical outcomes for patients with metastatic or recurrent MBC, large, comprehensive, real-world cohort studies are urgently warranted.

## Data Availability

The original contributions presented in the study are included in the article/supplementary material. Further inquiries can be directed to the corresponding authors.

## References

[1] Giordano SH . Breast cancer in men. N Engl J Med. (2018) 378:2311–20. doi: 10.1056/NEJMra1707939, PMID: 29897847

[2] Miao H Verkooijen HM Chia KS Bouchardy C Pukkala E Larønningen S . Incidence and outcome of male breast cancer: an international population-based study. J Clin Oncol. (2011) 29:4381–6. doi: 10.1200/JCO.2011.36.8902, PMID: 21969512

[3] Marczyk M Kahn A Silber A Rosenblit M Digiovanna MP Lustberg M . Trends in breast cancer-specific death by clinical stage at diagnoses between 2000 and 2017. J Natl Cancer Inst. (2025) 117:287–95. doi: 10.1093/jnci/djae241, PMID: 39348186

[4] Fang W Huang Y Han X Peng J Zheng M . Characteristics of metastasis and survival between male and female breast cancer with different molecular subtypes: A population-based observational study. Cancer Med. (2022) 11:764–77. doi: 10.1002/cam4.4469, PMID: 34898007 PMC8817100

[5] Ibragimova MK Tsyganov MM Kravtsova EA Tsydenova IA Litviakov NV . Organ-specificity of breast cancer metastasis. Int J Mol Sci. (2023) 24:15625. doi: 10.3390/ijms242115625, PMID: 37958607 PMC10650169

[6] Li S Li C Shao W Liu X Sun L Yu Z . Survival analysis and prognosis of patients with breast cancer with pleural metastasis. Front Oncol. (2023) 13:1104246. doi: 10.3389/fonc.2023.1104246, PMID: 37197429 PMC10183576

[7] Ohta Y Shimizu Y Matsumoto I Watanabe G . Management of Malignant pleural effusion by multimodality treatment including the use of paclitaxel administered by 24-hour intrathoracic infusion for patients with carcinomatous pleuritis. J Exp Clin Cancer Res. (2006) 25:15–9. 16761613

[8] Liu L Zhou Q Che G Wu Z Kou Y Li D . Surgical treatment of lung cancer by video-assisted thoracoscopic surgery. Zhongguo Fei Ai Za Zhi. (2004) 7:431–3. doi: 10.3779/j.issn.1009-3419.2004.05.13, PMID: 21244798

[9] Greif JM Pezzi CM Klimberg VS Bailey L Zuraek M . Gender differences in breast cancer: analysis of 13,000 breast cancers in men from the National Cancer Data Base. Ann Surg Oncol. (2012) 19:3199–204. doi: 10.1245/s10434-012-2479-z, PMID: 22766989

[10] Xie J Ying YY Xu B Li Y Zhang X Li C . Metastasis pattern and prognosis of male breast cancer patients in US: a population-based study from SEER database. Ther Adv Med Oncol. (2019) 11:1758835919889003. doi: 10.1177/1758835919889003, PMID: 31798694 PMC6859799

[11] Kreisman H Wolkove N Finkelstein HS Cohen C Margolese R Frank H . Breast cancer and thoracic metastases: review of 119 patients. Thorax. (1983) 38:175–9. doi: 10.1136/thx.38.3.175, PMID: 6857580 PMC459514

[12] Sanguinetti A Polistena A Lucchini R Monacelli M Galasse S Avenia S . Male breast cancer, clinical presentation, diagnosis and treatment: Twenty years of experience in our Breast Unit. Int J Surg Case Rep. (2016) 20s:8–11. doi: 10.1016/j.ijscr.2016.02.004, PMID: 26994487 PMC4883054

[13] Hogan KO Fan F . Diagnosis of metastatic adenoid cystic carcinoma of the breast on pleural fluid cytology in a 60-year-old male. Diagn Cytopathol. (2021) 49:E172–e74. doi: 10.1002/dc.24636, PMID: 33035408

[14] Huang HC Hang JF Wu MH Chou TY Chiu CH . Lung adenocarcinoma with ipsilateral breast metastasis: a simple coincidence? J Thorac Oncol. (2013) 8:974–9. doi: 10.1097/JTO.0b013e31828f6873, PMID: 23774384

[15] Friedel G Pastorino U Ginsberg RJ Goldstraw P Johnston M Pass H . Results of lung metastasectomy from breast cancer: prognostic criteria on the basis of 467 cases of the International Registry of Lung Metastases. Eur J Cardiothorac Surg. (2002) 22:335–44. doi: 10.1016/S1010-7940(02)00331-7, PMID: 12204720

[16] Hassett MJ Somerfield MR Baker ER Cardoso F Kansal KJ Kwait DC . Management of male breast cancer: ASCO guideline. J Clin Oncol. (2020) 38:1849–63. doi: 10.1200/JCO.19.03120, PMID: 32058842

[17] Welter S Jacobs J Krbek T Tötsch M Stamatis G . Pulmonary metastases of breast cancer. When is resection indicated? Eur J Cardiothorac Surg. (2008) 34:1228–34. doi: 10.1016/j.ejcts.2008.07.063, PMID: 18824371

[18] Cardoso F Paluch-Shimon S Senkus E Curigliano G Aapro MS André F . 5th ESO-ESMO international consensus guidelines for advanced breast cancer (ABC 5). Ann Oncol. (2020) 31:1623–49. doi: 10.1016/j.annonc.2020.09.010, PMID: 32979513 PMC7510449

[19] Palma DA Olson R Harrow S Gaede S Louie AV Haasbeek C . Stereotactic ablative radiotherapy versus standard of care palliative treatment in patients with oligometastatic cancers (SABR-COMET): a randomised, phase 2, open-label trial. Lancet. (2019) 393:2051–58. doi: 10.1016/S0140-6736(18)32487-5, PMID: 30982687

[20] Planchard D Soria JC Michiels S Grunenwald D Validire P Caliandro R . Uncertain benefit from surgery in patients with lung metastases from breast carcinoma. Cancer. (2004) 100:28–35. doi: 10.1002/cncr.11881, PMID: 14692021

[21] Rena O Papalia E Ruffini E Filosso PL Oliaro A Maggi G . The role of surgery in the management of solitary pulmonary nodule in breast cancer patients. Eur J Surg Oncol. (2007) 33:546–50. doi: 10.1016/j.ejso.2006.12.015, PMID: 17267164

[22] Tanaka F Li M Hanaoka N Bando T Fukuse T Hasegawa S . Surgery for pulmonary nodules in breast cancer patients. Ann Thorac Surg. (2005) 79:1711–4; discussion 14-5. doi: 10.1016/j.athoracsur.2004.10.033, PMID: 15854960

